# Complete mitochondrial genome sequence and phylogenetic analysis of *Arion ater* (Stylommatophora: Arionidae)

**DOI:** 10.1080/23802359.2021.1972862

**Published:** 2021-09-13

**Authors:** Xiaoyang Wu, Xibao Wang, Yongquan Shang, Guolei Sun, Qinguo Wei, Honghai Zhang

**Affiliations:** College of Life Science, Qufu Normal University, Qufu, Shandong, PR China

**Keywords:** *Arion ater*, mitochondrial genome, phylogenetic tree

## Abstract

*Arion ater* are a group of mollusks, almost all of which live in a dark and humid environment during their life cycle. In this study, the complete mitogenome of *Arion ater* was sequenced using next generation sequencing, analyzed and compared with other Stylommatophorans. The total length of the mitochondrial genome is 14,314 bp and consists of 13 protein-coding genes, 22 tRNA genes, 2 rRNA genes, and 1 control region. The genome has a typical mitochondrial gene sequence of *Arion*. Phylogenetic analysis of the mitochondrial genomes of 27 species of Stylommatophora showed that the *Arion ater*, *Arion rufus* and *Arion vulgaris* forms a monophyletic group with other species.

*Arion ater* (Stylommatophora: Arionidae), known as a slug, is hermaphrodite, and can reproduce by interfertilization (Wright 1974). The eggs of *Arion ater* are produced in the soil crevices with high humidity and concealment. The eggs lay in the crevices of the soil with high humidity and hidden (Wright 1974). *Arion ater* are long shuttle type, soft, smooth and without shell (Ross et al. 2017). The body surface is dark black, and the body surface is moist with mucus. There are two pairs of antennae (the front antennae and the posterior antennae) and horny teeth and tongues in the oral cavity. *Arion ater* like to live in a dark and humid environment and feed on a wide range of plants, such as taro, bread tree and other plants, fungi and their fruits (South 1992).

In this study, the complete mitochondrial genome *Arion ater* was sequenced and analyzed, which is helpful to identify the species and locate the evolutionary relationship of *Arion ater*, so that it can be used from a biological perspective. *Arion ater* samples used in this study were collected from a suburban area in North Cheshire (53.391463N, 2.211214W) (Joynson et al. 2017). The information of the DNA of *Arion ater* was deposited at the institute of protection and utilization for biological resource, Qufu Normal University (www.qfnu.edu.cn/, Xiaoyang Wu, wuxiaoyang1988@126.com) under the voucher number IPUBR-01. The mitochondrial genome of *Arion ater* was sequenced and assembled using Illumina high-throughput sequencing technology and NOVOPlasty 3.7.2 version software (Dierckxsens et al. 2017). MITOS were used to annotate the mitochondrial genome (Bernt et al. 2013). The preliminary results were compared with the protein-coding and ribosomal RNA of the mitochondrial genome of related species (*Arion vulgaris*, *Arion rufus*) using BLAST methods. After annotated, the mitochondrial genome was deposited in GeneBank with the accession number MW927710.

The total length of the mitochondrial genome is 14,314 bp, including a control region and contains 22 transfer RNA (tRNA) genes, two ribosomal RNA (rRNA) (12S and 16S rRNA) genes and 13 protein-coding genes (PCGs). Most of genes (9 PCGs, 14 tRNAs, and 1 rRNA) are encoded on the heavy (+) strand, while the remaining genes (nd3, cox3, ATP6, ATP8, 12S rRNA, tRNA-Arg, tRNA-Asn, tRNA-Glu, tRNA-Gln, tRNA-Leu, tRNA-Met, tRNA-Ser, tRNA-Thr) are located on the light (−) strand. The total composition of the sequence is 31.70% A, 14.64% C, 37.56% T and 16.08% G, and the content of *A* +* T* (69.27%) is higher than that of *C + G* (30.72%). All tRNA genes except three tRNA (tRNA-Ser (TCA, AGC), tRNA-Lys (AAA), tRNA-Cys (TGC)) could be folded into a typical cloverleaf structure with a length of 56–68 bp. 16s RNA and 12s RNA genes were located between tRNA-Val and tRNA-Leu and between tRNA-Gln and the D-loop, respectively. The initiation codons of 13 protein-coding genes were ATN codons except for *cox1*, *Cytb* and *nd5*. The stop codons of the protein-coding genes were TAN except for *cox3*.

Despite their great diversity and relevance, the internal phylogeny of Stylommatophora has been debated (Xie et al. 2019). We downloaded 27 complete mitochondrial genomes of related species from NCBI. The maximum-likelihood (ML) method was used to construct a phylogenetic tree and the bootstrap value was set to 1000 using MEGA 7 software (https://www.megasoftware.net/). The Bayesian Inference (BI) method was also used to construct a phylogenetic tree and the generations value was set to 100,000 using MrBays software (http://nbisweden.github.io/MrBayes/). The phylogenetic trees generated from ML and BI methods have the same topologies (Figure 1) and two major phyletic lineages were present in Stylommatophora. A well-supported sister group relationship between Arionoidea and Philomycidea was recovered. The taxonomic position of *A. ater* is sister species to *A. rufus* and *A. vulgaris* and all of the three species belong to Arionidae (Figure 1). Furthermore, the phylogenetic relationship of Arionidae, Philomycidae and another genus belong to Stylommatophora were controversial in the past, and the discussion were focused on phylogeny and divergence times of stylommatophoran species (Doğan et al. 2020). We expect the data of present study to provide a useful for further research and phylogenetic relationship of Stylommatophora.

**Figure 1.**
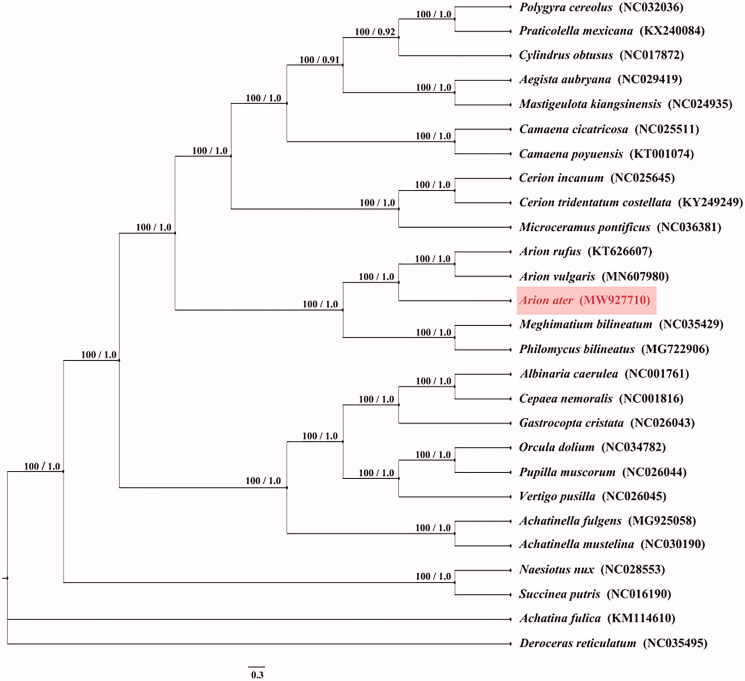
Phylogenetic tree of 27 species was obtain from maximum-likelihood (ML) and Bayesian phylogenetic inference (BI) method based on all protein-coding genes, the ML bootstrap proportions and BI posterior probabilities are shown on the nodes. Other mitochondrial genomes were downloaded from the NCBI Nucleotide databases (www.ncbi.nlm.nih.gov/nuccore).

## Data Availability

The genome sequence data that support the findings of this study are openly available in GenBank of NCBI (https://www.ncbi.nlm.nih.gov) under the accession no. MW927710. A total of sequences were downloaded from GenBank (http://www.ncbi.nlm.nih.gov/). The associated BioProject, SRA, and Bio-Sample numbers are PRJEB21599, ERS1807597, and SAMEA104148579, respectively.
